# Is Silver the New Gold? A Systematic Review of the Preclinical Evidence of Its Use in Bone Substitutes as Antiseptic

**DOI:** 10.3390/antibiotics11080995

**Published:** 2022-07-24

**Authors:** Michele Fiore, Alessandro Bruschi, Claudio Giannini, Lorenzo Morante, Claudia Rondinella, Matteo Filippini, Andrea Sambri, Massimiliano De Paolis

**Affiliations:** Orthopaedic and Traumatology Unit, IRRCS Azienda Ospedaliera-Universitaria di Bologna, 40138 Bologna, Italy; alessandro.bruschi@ior.it (A.B.); claudio.giannini@ior.it (C.G.); lorenzo.morante@ior.it (L.M.); claudia.rondinella@ior.it (C.R.); matteo.filippini@ior.it (M.F.); andrea.sambri2@unibo.it (A.S.); massimiliano.depaolis@aosp.bo.it (M.D.P.)

**Keywords:** bone and joint infections, orthopaedic, odontology, bone substitutes, silver compounds, silver ions, silver nanoparticles

## Abstract

Antibiotic-laden bone substitutes represent a viable option in the treatment of bone and joint infections with bone defects. In particular, the addition of silver ions or silver nanoparticles to bone substitutes to achieve local antiseptic activity could represent a further contribution, also helping to prevent bacterial resistance to antibiotics. An in-depth search of the main scientific databases was performed regarding the use of silver compounds for bone substitution. The available evidence is still limited to the preclinical level: 22 laboratory studies, 2 animal models, and 3 studies, with both in vitro and in vivo analysis, were found on the topic. Numerous biomaterials have been evaluated. In vitro studies confirmed that silver in bone substitutes retains the antibacterial activity already demonstrated in coatings materials. Cytotoxicity was generally found to be low and only related to silver concentrations higher than those sufficient to achieve antibacterial activity. Instead, there are only a few in vivo studies, which appear to confirm antibacterial efficacy, although there is insufficient evidence on the pharmacokinetics and safety profile of the compounds investigated. In conclusion, research on bone substitutes doped with silver is in its early stages, but the preliminary findings seem promising.

## 1. Introduction

Bone and joint infections (BJIs) represent an extremely heterogeneous group of diseases, including implant-associated infections (both in the fields of orthopaedics and odontology), septic arthritis, and osteomyelitis [1]. The increase in BJIs’ incidence shown in recent years, mainly due to the increase in joint replacements and the use of orthopaedic hardware, currently represents a growing social and economic issue for health systems [1,2]. Indeed, implant-related infection rate is reported to be 5% for primary cases, 6% for revision cases and increases to 43% for previously infected cases [3]. Osteomyelitis, defined as a bone inflammation caused by infection, may be the common endpoint of BJIs. Bacterial infections complicate the bone-healing process following fractures or surgical treatment, often resulting in significant bone loss [3]. Once occurred, bone infections are very challenging to treat, due to the difficulty of achieving a suitable antibiotic concentration in the affected area that may permit bacteria eradication [4]. Hence, the treatment of BJIs generally requires wide debridement with removal of all infected bone and soft tissues, irrigation, and, subsequently, dead-space filling [5,6]. Bone defects wider than 2 cm or circumferential losses involving more than 50% of bone are defined as critical-size bone defects (CSDs). CSDs usually progress to healing failure, even after optimal fixation [7]. In order to restore the continuity of the bone loss resulting from the surgical treatment, autologous, allogenic and artificial bone can be implanted [6,8]. Autograft substitutes are still considered the gold standard for bone repair and regeneration due to their osteogenic nature combined with no immunological side effects of the graft [7,9]; however, they are associated with donor site morbidity (hematomas, infection, and neurovascular injury) and longer operative time [10]. On the other hand, when using allografts, the principal concerns are related to mechanical resistance, limited osteoconduction and risk of infections [8]. Therefore, greater attention has increasingly been given to bone graft substitutes. They have been defined as “a synthetic or biologically organic combinations which can be inserted for the treatment of a bone defect instead of autogenous or allogenous bone” [10]. Theoretically, the bone substitutes mimic bone graft, combining advantages of natural and synthetic biomaterials [8,9] and supporting local bone healing [7]. The ideal bone substitute should be biocompatible, osteoconductive, osteoinductive, resorbable, thermally nonconductive, sterilizable, and available at a reasonable cost [10]. Bone substitutes can be broadly categorized into ceramics (nonresorbable and biodegradable), hydroxyapatite, β-tri-calcium phosphate, calcium sulfate, calcium carbonate, silicate (“Bioglass”), magnesium composites and calcium phosphate cements [7]. Current advances have been made with the development of tissue-engineered products, incorporating growth factors and stem cells [10].

Despite the development in biomaterials’ properties, risk of infection remains a major issue after implantation. Thus, antibacterial properties should be considered during the development and choice of a bone substitute, as well as biocompatibility and physic-chemical features [11,12]. This is in order to prevent infections of the bone substitutes, or to optimise their use in case of BJIs. In fact, the result of bacterial adhesion to implants or grafts usually progresses with complete removal [12]. Moreover, to prevent infection recurrence, systemic or local antibiotics should be administrated after surgery; nevertheless, inappropriate and excessive use of antibiotics, in addition to systemic side effects for the patient, increases the risk of the emergence of multidrug-resistant bacteria [6]. The addition of antimicrobial nanomaterials, such as silver, zinc, copper, carbon nanotube, graphene oxide, molybdenum disulfide and titanium oxide, into the biomaterials has significantly shown to inhibit microbial infection, determining also a lower tendency to develop bacterial resistance [9,13,14]. 

In particular, silver’s efficacy and safety has been reported in several in vitro and animal studies [15]. Silver ions (Ag) and silver nanoparticles (AgNPs) have garnered prominent consideration in recent years due to their broad spectrum of antibacterial properties, low bacterial resistance, and relatively low cytotoxicity [13]. For these reasons, the use of silver gained interest in the clinical practice with applications such as wound healing and cardiac and orthopaedic implant coating. Particularly in the orthopaedic field, the use of silver has proved to be effective in the treatment of megaprosthesis infections [15]. 

Although only preclinical studies about the use of silver combined with bone substitutes have been published, this issue may have important clinical implications for the prevention and treatment of BJIs. The aim of this review is to provide an overview of the evidence currently available in the literature.

## 2. Results

A total of 271 studies were found through the electronic search and 8 studies were added after cross-referenced research on the bibliography of the examined full-text articles. After a preliminary analysis, a total of 27 studies were included in this systematic review [3,4,6,8,9,11,12,13,16,17,18,19,20,21,22,23,24,25,26,27,28,29,30,31,32,33,34]. To date, there are no clinical studies on the use of bone substitutes containing adjuvant silver. Twenty-two laboratory studies [4,6,8,9,11,12,13,16,17,18,19,20,22,23,24,25,26,27,28,29,30,31,32,34], two animal models [21,33] and three studies in which both in vitro and in vivo analysis were performed [3,6,31] were found on the topic. 

A wide range of biomaterials were evaluated as possible carriers of silver in bone. The tests used to appraise the in vitro or in vivo antibacterial activity of the compounds in the various studies included: cultures from bone samples, agar diffusion, halo test, agar dilution, broth microdilution, spread plate method, bacterial count through scanning electron microscope, confocal laser scanning microscopy, epi-fluorescence microscopy, histopathological examinations, RT-PCR bacterial DNA measurement, and radiological examination. All in vitro studies reported a partial or total inhibition of the bacterial growth. All in vivo studies that directly investigated the antibacterial effect of silver compounds confirmed their efficacy in the treatment of osteomyelitis. The data on cell and tissue toxicity are inconsistent; however, in all the studies, the antibacterial activity of the compounds tested was reported at nontoxic concentrations. Extended data from the included studies are reported in Table 1.

## 3. Discussion

Silver antimicrobial activity relies on several mechanisms. Principally, it stops cells’ respiratory chain, affecting the cells’ energy generation, due to its affinity to the sulfhydryl and thiol groups [35]. Additionally, silver leads to a release of potassium [36], binds DNA and RNA, disrupting the cells’ translation and transcription processes [37], and produces intracellular reactive oxygen species [38]. Consequently, silver has the ability to eliminate a broad spectrum of pathogens that can be found at implant sites [39]. Surfaces or materials containing silver particles act by directly releasing ions into the water solution in which they lie. More recently, silver technology has focused on the use of nanoparticles. AgNPs seems to be more reactive, with a stronger antibiofilm potential than their bulk metal counterparts, partially due to the increased active surface area [40,41]. AgNPs are usually 1 nm to 20 nm in size. Because of their small dimensions, the surface area is taken advantage of, passing more into cell membranes, thereby contributing to augmented antimicrobial action [42]. Furthermore, antibacterial mechanisms of AgNPs have been hypothesized that do not depend on the release of ions but are related to the interaction between silver and other substrates. For example, interaction with some titanium alloys can lead to the production of an electron cloud around the surface of the compounds [35]. This cathodic reaction, which produces a proton depletion region, would appear to reduce the transmembrane proton electrochemical gradient and lead to bacterial death by interfering with ATP synthesis [35]. In addition, to date, there is limited evidence that AgNPs possess osteoconductive capabilities, promoting the proliferation of mesenchymal stem cells and their osteogenic differentiation in vitro, as well as enhancing bone fracture healing in animal models [43].

The clinical use of silver for antibacterial purposes in implantable devices has been mainly investigated as a coating material [15]. Therefore, there is already evidence regarding the efficacy and safety profile of this application. Studies largely agree on time and concentration influencing the bactericidal effect of silver in bone substitutes, with higher Ag concentration and longer exposure time associated with better antibacterial responses [3,22]. Accordingly, a cytotoxic effect of silver at excessively high concentrations is to be expected, by means of the same antibacterial mechanisms, to which, however, eukaryotic cells are less sensitive at low concentrations because of their extremely better antioxidant and DNA repair activity [44]. It has been reported that silver has toxic effects for humans at a high blood concentration of 300–1200 ppb [45]. Instead, silver concentrations below 200 ppb can be considered as normal [37]. Instead, regarding AgNPs’ safety profile, various studies suggested that there is a large gap, at least by an order of magnitude, between the toxic and antibacterial doses of AgNPs [46,47]. All available clinical data on the pharmacokinetics of silver are related to its use in coating materials, especially regarding serum levels, which have never been found to exceed the threshold of toxicity. Nevertheless, several side effects have been related to silver in clinical studies, including diffuse argyria, kidney and liver damage, leukopenia, and peripheral neuropathies [36,48,49]. No embryotoxic side effects in humans are described [50]. 

In contrast to silver used in coatings, this review highlighted that there are currently no clinical data on the use of silver as an adjuvant in biomaterials for bone substitution. However, there are some in vivo studies using animal models and a substantial number of laboratory studies investigating the antibacterial efficacy of silver on a plethora of different biomaterials. 

All in vitro studies included in this review supported that even when used as a biomaterial constituent, silver appears to preserve its antibacterial activity [3,4,6,8,9,11,12,13,16,17,18,19,20,22,23,24,25,26,27,28,29,30,31,32,34]. This evidence emerged regardless of the type of analysis used by individual studies to highlight the effect of silver. In particular, the impact of silver was found to be cross-cutting for both Gram+ and Gram- bacteria. Some studies hypothesised that due to the different composition of the membranes, silver-doped materials could have more antibacterial effect against Gram+ [14,25,26]. However, other studies did not report differential antibacterial effect, showing a complete response in both cases [3,27,29]. Unfortunately, no study included in this review conducted quantitative evaluations specifically aimed at investigating this aspect using different bacterial species clustered according to membrane characteristics. Three out of five studies with animal models directly investigated the in vivo antibacterial effect of silver compounds. All confirmed the efficacy of using biomaterials containing adjuvant silver in the treatment of S. aureus osteomyelitis [21,31,33]. In detail: (1) Zhang et al. investigated silver application in two hydroxyapatite compounds with different silver concentrations [33]; (2) calcium phosphate beads doped with silver ions were used in the study by Kose et al. [21]; (3) Weng et al. instead considered the use of silver nanoparticle (AgNP)-loaded nano-hydroxyapatite-reduced graphene oxide scaffolds [31]. In all in vivo studies, it was also confirmed that the presence of silver did not affect the osteoconductive and osteoinductive activity of the bone substitutes employed [3,6,21,31,33].

The ever-increasing resistance of human pathogens to antibiotics makes silver a convenient alternative to be harnessed as an antibacterial weapon. Indeed, bacterial resistance hardly arises in the presence of silver ions in the environment [51,52], while no relevant data describing bacterial resistance to AgNPs have been reported yet [53]. Therefore, it would be interesting to examine the antibacterial effect of silver compared with antibiotics in bone substitutes. However, only few conflicting data are available: Kose et al. showed a better result for silver compared to vancomycin in murine models [21]; on the other side, two studies showed a better antibacterial effect of bone substitutes doped with doxycycline or gentamicin alone [22,27].

Little can be stated about the safety profile of silver use in biomaterials. Indeed, it might be expected that the pharmacokinetics of silver used as a constituent material in bone substitutes would differ radically from its use in coatings. This could lead to the ineffective release of silver in vivo, exceeding toxicity thresholds, or even irregular or excessively phasic kinetics that alternate between these two possibilities. Several in vitro studies have evaluated the kinetics of silver release from biomaterials in water or in different solutions of simulated body fluid. However, on the one hand, the extreme heterogeneity of the materials tested, and on the other hand, the radical differences in environment compared to a real setting, fully restrict the deducible conclusions. In fact, only in vivo assessments may allow approximating the pharmacokinetics. Similar remarks can be made about cytotoxicity from in vitro studies, with results varying widely due to the broad spectrum of materials analysed. However, in all in vitro studies, the antibacterial activity of the compounds analysed was achieved at noncytotoxic concentrations. From this point of view, with regard to the in vivo studies, Zhang et al. found that the silver concentration of the bone tissue was found to be over 2 ppm in the n-HA/PU10 group with local toxic risk increasing when the silver concentration exceeds 1 ppm in tissue, as recommended by the safety guidelines [33]. Moreover, the same compound has shown the possibility of inducing liver toxicity [33]. In contrast, the studies by Kose et al. and Yuan et al. found no local and systemic toxicity in the animal models used, both investigating calcium phosphate derivates doped with silver ions or nanoparticles, respectively [3,21]. Shimabukuro et al. reported that the inflammatory effect of silver phosphate (Ag_3_PO_4_) in a bone substitute composed of carbonate apatite (CO_3_Ap) was concentration-dependent [6]. Similarly, the study by Schneider et al., not included in this review as it did not directly investigate the antibacterial effect, showed no inflammatory reactions to cotton-wool-like silver-doped calcium phosphate nanocomposites in sheep [54]. Another study, not included in this review for the same reason, performed by Wnukiewicz et al., examined the soft-tissue reaction to corundum ceramic with colloidal silver in rabbits, finding no difference with the control group [55]. 

## 4. Materials and Methods

**Figure 1:**
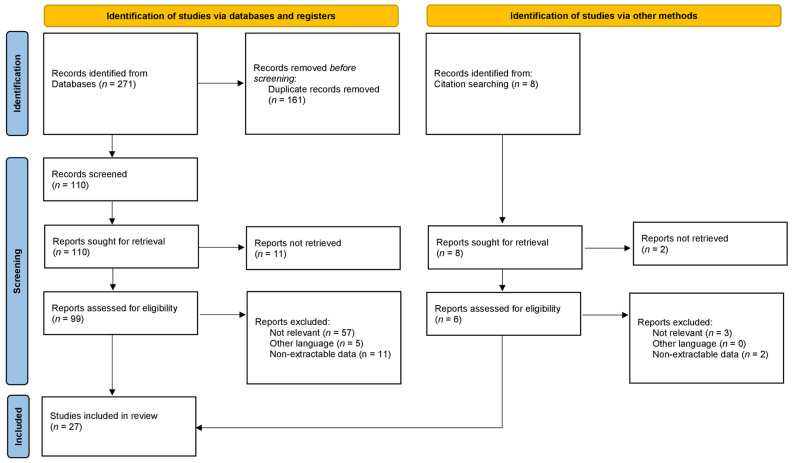
PRISMA 2020 flow diagram and the selection of studies.

An in-depth search of the scientific research was performed according to 2020 PRISMA (preferred reporting items for systematic reviews and meta-analyses) guidelines [56]. The search algorithm according to these guidelines is shown in Figure 1. 

A search regarding the existing evidence on the use of silver compounds and silver nanoparticles combined with biomaterials for bone substitution with no restriction on date of publication, up to the end of June 2022, was performed on the PubMed (https://pubmed.ncbi.nlm.nih.gov/ (accessed on 30 June 2022)), Scopus (https://www.scopus.com (accessed on 30 June 2022)), and Web of Science (www.webofscience.com (accessed on 30 June 2022)) databases. Various combinations of the following keywords were used: “silver compound”, “silver nanoparticles”, “bone substitutes”, “bone biomaterials”. The inclusion criteria were as follows: original research reporting preclinical results on in vitro testing and in vivo animal models of the antibacterial activity, pharmacokinetics and pharmacodynamics of silver combined with biomaterials used for bone substitution. Only studies in English were retained. Articles that were considered relevant during the electronic search were retrieved in full-text, and a cross-referencing hand-search of their bibliography was performed, in order to find further related articles. Reviews and meta-analysis were also analysed, in order to broaden the search for studies that might have been missed through the electronic search.

A formal assessment of the quality of the articles was not conducted, as there is no clear evidence of validated tools for the evaluation of preclinical laboratory studies. Only descriptive statistics were used for this study as the type of data provided.

The following data were independently extracted by all the investigators and summarized in Table 1: study type, material tested, type of antimicrobial activity evaluations, microorganisms tested, and findings about toxicity. 

## 5. Conclusions

The introduction of bone substitutes doped with ionic silver or silver nanoparticles into clinical practice would provide a valuable further contribution to the management of challenging diseases such as osteomyelitis and peri-prosthetic or implant-related infections, as well as to the prevention of bacterial resistance to antibiotics. Numerous materials have already been evaluated for this purpose, but the available evidence is still limited to the preclinical level. In vitro studies have confirmed that when silver is added to bone substitutes, it retains the antibacterial activity already demonstrated in coatings materials. The antibacterial effect against Gram+ might be higher than against Gram-. However, conclusive data are lacking as well as it is unclear whether silver could provide greater efficacy than antibiotic loading. The cytotoxicity of silver compounds has generally been shown to be low and only related to concentrations of silver significantly higher than those sufficient to achieve antibacterial activity. On the other hand, there are only a few in vivo studies which appear to confirm antibacterial efficacy, although there is insufficient evidence on the pharmacokinetics and safety profile of the biomaterials investigated. In conclusion, research on bone substitutes doped with silver is in its early stages but the preliminary findings seem promising.

## Figures and Tables

**Table 1:** Data from included studies.

Study	Type of Study	Material Tested	Antimicrobial Activity Evaluations	Bacteria Tested	Reported Results	Toxicity
Afzal, 2012 [12]	In vitro	Hydroxyapatite–silver (Ag-HA) and carbon nanotube–silver (CNT-Ag) composites	Bacterial count through SEM	*Escherichia coli* *Staphylococcus epidermidis*	Partial response.	N/A
Bee, 2020 [13]	In vitro	Antibacterial silver-nanoparticle-decorated hydroxyapatite (HAp/AgNP)	Agar diffusion	*Staphylococcus aureus*	Zone of inhibition of bacterial growth.	N/A
Bostancıoğlu, 2015 [11]	In vitro	Silver-doped calcium-phosphate-based inorganic powder (ABT)	Agar diffusion Agar dilution	*Escherichia coli* *Pseudomonas aeruginosa* *Staphylococcus aureus*	Partial response or total response depending on dilution and concentration.	Concentration-dependent cytotoxicity on V79 379A and HUVEC lines. ABT is noncytotoxic and bears good biocompatibility even at 1000 μg mL^−1^ of ABT with the highest content of silver.
Correia, 2016 [8]	In vitro	Tricalcium phosphate (TCP)/sodium alginate scaffold doped with AgNP	Agar diffusion	*Staphylococcus aureus*	Halo of 0.820 cm with 1 cm scaffold.	No cytotoxicity on osteoblast cells.
Dalavi, 2020 [9]	In vitro	Alginate-nanohydroxyapatite doped with chitooligosaccharide-coated silver nanoparticles (COS-Ag-Alg-HA)	Broth microdilution	*Staphylococcus aureus*	Total response at higher concentration than 77.2% using 3 mg/mL of microsphere.	No cytotoxicity on human osteosarcoma osteoblast-like MG-63 cells.
Deng, 2017 [16]	In vitro	PEEK doped with Ag + nanoparticles	Agar diffusion	*Staphylococcus aureus* *Escherichia coli*	Halo of 14 mm of inhibition for both the bacteria with 0.9 mm scaffold.	Initial low proliferation rate of human osteosarcoma osteoblast-like MG-63 cells.
Gong, 2017 [17]	In vitro	Silver-doped hydroxyapatite (Ag-HA) + Bio-Oss	RT-PCR bacterial DNA measurement	*Porphyromonas gingivalis* *Fusobacterium nucleatum*	Partial response, with decreasing of bacterial DNA at 2 h, 4 h, and 24 h compared to control group in which no inhibition was seen.	AgHA showed obvious cytotoxicity against periodontal fibroblasts and rat bone-marrow stromal cells, with relative survival rates of <80%. Bio-Oss only showed survival rates exceeding 95% of periodontal.
Jacquart, 2013 [18]	In vitro	Calcium carbonate–calcium phosphate bone cement doped with silver (Ag-CaCO_3_-CaP)	Broth microdilution	*Staphylococcus aureus* *Escherichia coli*	Complete response.	No cytotoxicity on human bone marrow stroma cells.
Jegatheeswaran, 2015 [19]	In vitro	Polyethylene-glycol/hydroxyapatite doped with silver (Ag-HAp-PEG)	Epi-fluorescence microscopy	*Escherichia coli*	Partial response with increasing bacteria death in analyses at 6 and 12 h.	N/A
Jiang, 2016 [20]	In vitro	Hydroxyapatite/polyurethane composite scaffolds doped with silver phosphate particles (Ag_3_PO_4_-n-HA/PU)	Agar diffusion	*Staphylococcus aureus* *Escherichia coli*	The bacteriostatic rate resulted time and weight percentage of Ag incorporated depending.	Scaffolds with no more than 5 wt% appear to have no cytotoxicity on human osteosarcoma osteoblast-like MG-63 cells. Higher concentration (>5%) would weaken cytocompatibility.
Kose, 2020 [21]	In vivo (rabbit)	Calcium phosphate (CP) with silver ions	Radiological examination Bacterial cultures from bone samples Histopathological examinations	*Staphylococcus aureus*	No MRSA was found at cultures, no X-ray signs of osteomyelitis and no sign of chronic inflammation in histological analysis, compared to the control groups.	No inflammatory reactions.
Sampath Kumar, 2015 [22]	In vitro	Calcium-deficient hydroxyapatite (CDHA) carrier of doxycycline and Ag+ ions	MIC/MBC studies and time-kill assay	*Staphylococcus aureus* *Escherichia coli*	When compared with doxycycline, the antibiotic release provided the initial high antibacterial activity, while the sustained ion release provided a long-term antibacterial activity.	No cytotoxicity on L6 myoblast cells.
Lim, 2014 [23]	In vitro	Silver and silicon-containing apatite (Ag,Si-HA)	Bacterial count through SEM	*Staphylococcus aureus* *Escherichia coli*	No bacteria growth compared to negative control: complete response.	MSCs treated with Ag,Si-HA showed an initial low proliferation rate compared to controls, and faster proliferation after day 3.
Nam, 2017 [24]	In vitro	Portland cement doped with silver nanoparticles (SNPC)	Agar diffusion	*Streptococcus mutans* *Streptococcus sobrinus*	1.0% wt of SNPC has no antibacterial effect; 3.0 wt% SNPC inhibited *S. sorbinus* by 1.9 ± 0.5 mm, while no inhibition halos were shown for *S. mutans* at the same dose. SNPC of 5.0 wt% significantly inhibited S. *sorbinus* (halo diameter 4.2 ± 0.3 mm) and *S. mutans* (halo diameter 2.2 ± 0.4 mm).	N/A
Paterson, 2020 [4]	In vitro	Polycaprolactone scaffolds with silver-doped hydroxyapatite (Ag-nHA)	Agar diffusion	*Staphylococcus aureus* *Escherichia coli*	The scaffold reduced the viable bacteria count to undetectable levels by 48 h for *E. coli* and 96 h for *S. aureus*: complete response.	Silver-doped nHA to enhance MSC differentiation down an osteogenic path. Scaffolds containing 10 mol.% silver may be toxic for MSCs.
Sethmann, 2018 [25]	In vitro	Phosphatized Calcium Carbonate biomineral (PCCB) doped with Ag + silver ions	Agar diffusion	*Pseudomonas aeruginosa* *Staphylococcus aureus*	Samples treated with an AgNO3 solution with 10 mmol/L showed nearly the same antibacterial performance as samples treated with 100 mmol/L. Halo of 1.1–1.2 mm for Gram- and 3 mm for Gram+.	N/A
Shimabukuro, 2021 [6]	In vitro + in vivo (rabbit)	Silver phosphate in carbonate apatite (Ag_3_PO_4_-CO_3_Ap)	Agar diffusion immunofluorescence	*Staphylococcus epidermidis*	Antibacterial effect if concentration of Ag3Po4 is more than 0.1 wt %. Complete response.	Ag_3_PO_4_ content of 0.1–0.95 wt % may show antibacterial properties without cytotoxicity. Higher concentrations showed increasing toxicity for MC3T3-E1 cells. Ag_3_PO_4_ content of 0.1–0.3 wt % in the samples did not affect bone formation in vivo.
Sonamuthu, 2018 [26]	In vitro	Fluorinate-hydroxyapatite/polyvinyl alcohol doped with silver nanoparticles (AgNp-fHA)	Agar diffusion CLSM Broth microdilution	*Staphylococcus aureus* *Escherichia coli*	Antibacterial activity is time- and concentration-dependent. More effect on Gram + due to the different composition of membrane; complete response G+ and G- partial response in CLSM.	No cytotoxicity on human osteosarcoma osteoblast-like MG-63 cells.
Sowmya-Srinavasan, 2013 [27]	In vitro	Bioactive alpha- and beta-chitin hydrogel/nanobioactive glass ceramic doped with silver	Agar diffusion	*Staphylococcus aureus* *Escherichia coli*	Antibacterial activity of Ag dose dependent, similar effect between G+ and G-, but less effective than gentamicin alone.	No cytotoxicity on human primary osteoblasts and human periodontal ligament cells.
Verné, 2009 [29] + Miola, 2009 [28]	In vitro	SiO-CaO-NaO-AlO doped with silver (Ag-SCNA)	Agar diffusion Broth microdilution	*Staphylococcus aureus* *Escherichia coli*	Same antimicrobial activity against G+ and G-, halo of 2 mm.	No cytotoxicity on fibroblasts. Slightly lower proliferation rate compared to control cells.
Vollmer, 2016 [30]	In vitro	Calcium phosphate (CaP) doped with silver	Agar diffusion Bacterial count through SEM	*Escherichia coli*	Antimicrobial activity with halo in agar diffusion (no dimensions reported) and characteristics of poor health of bacteria at SEM compared to control.	No cytotoxicity on human osteoblasts.
Weng, 2020 [31]	In vitro + in vivo (rabbit)	Loaded nano-hydroxyapatite-reduced graphene oxide doped with Ag nanoparticles (AgNp-AHRG)	Agar diffusion Kirby–Bauer diffusion WBC count CRP Radiological examination	*Staphylococcus aureus*	Antibacterial activity in vitro and the halo zone is dependent on the concentration of Ag. In vivo, it significantly reduced the levels of inflammatory markers, such as leukocytes and CRP, after implantation in the infected site. In subsequent observations, the healing of the bone in the implanted group was significantly improved compared to the untreated group.	Concentration-dependent cytotoxicity on bone marrow stromal cells. No cytotoxicity for 1% and 2% silver AgNp-AHRG scaffolds.
Wilcock, 2017 [32]	In vitro	Hydroxyapatite paste silver doped (Ag-nHA)	Agar diffusion	*Pseudomonas aeruginosa* *Staphylococcus aureus*	Antibacterial activity dependent on Ag concentration.	N/A
Yuan, 2016 [3]	In vitro + in vivo (rabbit)	Porous β-tricalcium phosphate with Ag nanoparticles (AgNp- βTCP)	Agar diffusion Bacterial count through SEM	*Staphylococcus aureus* *Escherichia coli*	Antibacterial activity dependent on concentration. Difference in activity between G+ and G- was not reported. At SEM, there is some bacteria visible, but no biofilm was seen.	No local and systemic toxicity.
Zhang, 2019 [33]	In vivo (rabbit)	Nano-hydroxyapatite/polyurethane composite scaffolds doped with silver phosphate particles (Ag/n-HA/PU)	WBC count Radiological examination Histopathological examinations	*Staphylococcus aureus*	Radiological healing of infection with no difference between 3% wt and 10% wt concentration as well as no difference in histological analysis for trabeculae formation.	Local toxicity for highest concentration of silver (Ag/n-HA/10PU).
Zhang, 2020 [34]	In vitro	Brushite/Ag3PO4-coated Mg-based scaffolds (Mg-DCPD-Ag)	Spread plate method Bacterial count through SEM	*Staphylococcus aureus* *Escherichia coli* *Staphylococcus epidermidis*	Antibacterial activity with complete response depending on concentration of Ag.	Cytotoxicity for highest concentration of silver (Mg-DCPD-0.46 Ag)

Abbreviations: SEM, scanning electron microscope; MRSA, methicillin-resistant Staphylococcus aureus; WBC, white blood cells; CLSM, confocal laser scanning microscopy; MSC, mesenchymal Stem Cell.

## Data Availability

The data reported in this study are available in the literature.
